# Community pharmacists as antimicrobial guardians and gatekeepers – A qualitative study of the perspectives of pharmacy sector stakeholders

**DOI:** 10.1016/j.rcsop.2022.100212

**Published:** 2022-12-13

**Authors:** Kathryn Lim, Alex Broom, Anna Olsen, Holly Seale

**Affiliations:** a School of Population Health, University of New South Wales, Sydney, Australia; b School of Social and Political Sciences, The University of Sydney, Australia; c Research School of Population Health, Australian National University, Canberra, Australia

**Keywords:** Antimicrobial stewardship, Antimicrobial resistance, Pharmacy, Pharmacists, Qualitative research

## Abstract

**Background:**

Community pharmacists, as primary care providers, are an underutilised resource in antimicrobial stewardship (AMS). Primary care plays an important role in tackling antimicrobial resistance (AMR) as the principle of balancing access to antimicrobials while ensuring optimal use is agnostic to health setting. Understanding the sector's perceptions and practices towards AMS involvement is a continuing focus area of research. However, there is an opportunity to understand the sociological factors which influence the profession's contribution to stewardship practice, particularly across a broader spectrum of sector stakeholders at the individual, practice, system, and policy levels.

**Objective:**

To explore stakeholders' perceptions of the Australian community pharmacy sector's AMS involvement.

**Methods:**

Semi-structured interviews were conducted with fifteen key informants from the Australian community pharmacy sector. Participants' insights were invited across three broad areas: (1) understanding of AMR and AMS; and the (2) current and (3) future state of community pharmacy's AMS involvement. Interviews were audio-recorded, transcribed verbatim and analyzed using a combined method of inductive (informed by the Theoretical Domains Framework) and deductive thematic analysis.

**Results:**

Perceptions on promoting community pharmacists' AMS involvement within their existing role in promoting the quality use of medicines were heard. Adopting an antimicrobial guardian or gatekeeper role was perceived as influenced by the timing of their interaction with a patient either prior to, or post-consultation with a general practitioner (GP). Suggestions that the profession's potential and actual role in AMS could be challenged or even delimited due to lack of access to completeness of clinical information, and perceived consequences from a clinical and professional engagement perspective were also heard.

**Conclusion:**

Collaborative partnerships between GPs and community pharmacists, framing stewardship within a quality use of medicines agenda, and highlighting connections between pharmacists' professional services such as minor ailments are key elements enabling community pharmacist's antimicrobial gatekeeper and guardian role.

## 1 Background

Australia is in the top 25% of countries prescribing antibiotics in primary care.^1^ In 2019, over 10 million people were reported as having at least one antimicrobial prescription dispensed in the community. Further, over 80% of patients diagnosed with acute bronchitis or acute sinusitis prescribed antibiotics despite this being contrary to the clinical treatment guidelines for these conditions.^2^ General practice (GP) has been a key focus in supporting primary care involvement in antimicrobial stewardship (AMS).^3^^,^^4^ This has included initiatives such as promoting clinical guidelines, regulatory interventions for antibiotic prescriptions, stewardship education and resources, and encouraging participation in awareness campaigns.^5^ However, findings by Saha and colleagues (2020) indicated that while Australian GPs were aware of the importance of AMS, most did not routinely adopt these evidence-based strategies because of factors such as the lack of implementation resources, system structures and facilities.^5^ This suggests that continued and sustained efforts to support GP involvement in AMS are required. Further, there is an opportunity to extend this focus beyond the prescriber, to involve a range of healthcare professionals, such as community pharmacists^6^ to mirror a multidisciplinary AMS approach promoted within hospitals in primary care.

Community pharmacists are an underutilised resource in AMS.^7^^,^^8^ Despite inherent interactions with consumers in managing and dispensing antimicrobial prescriptions, there remains a limited understanding of the pharmacy sector's perceptions and attitudes towards AMS involvement. AMS-related educational resources and awareness campaigns are increasingly targeting community pharmacists as a key audience and research suggests that Australian community pharmacists recognise the importance of AMS.^9^^,^^10^ However, lack of collaboration with GPs and poor uptake of AMS resources reflect the need for enhanced upskilling and involvement of community pharmacists in AMS.^9^^,^^10^

In order to better integrate community pharmacists into AMS systems and practices, the complex set of conditions which promote or mitigate AMR should be considered. This includes individuals' attitudes and behaviours as well as cultural and structural frameworks that facilitate or create barriers to implementation of new practices. This study explores the context of AMS and perceptions among a broad spectrum of stakeholders involved with the Australian community pharmacy sector to highlight areas for future development and to increase community pharmacists' involvement in optimising antimicrobial use.

## 2 Methods

This was an exploratory qualitative study using semi-structured interviews with key informants within the Australian community pharmacy sector.

### 2.1 Participant recruitment and setting

The study used a purposive sampling approach^11^ to recruit key informants for the study. Key informants were defined as people who have active engagement or perform related activities that interact either directly or indirectly with the Australian community sector and have a degree of knowledge of AMS. This included those in clinical practice, policy/program development or research.

Potential participants were identified using contacts known to the research team and publicly available information. This included governance committee information sourced from peak pharmacy body websites (*n* = 58), and a random search of community pharmacies (*n* = 156) in metropolitan, rural and regional locations as identified using their postcode details sourced through the health service search engine, healthdirect Australia.^12^ Potential participants were contacted by email between June 2019 and September 2021 with an invitation to participate and a study information sheet. The long duration of recruitment was a result of the impact of COVID-19 and the decision to postpone follow up during the earlier phases of the pandemic, given the pressures that the pandemic response had on health professionals. Of the 214 potential participants emailed, 15 consented, 7 declined with the most common response being capacity constraints, and 192 failed to respond.

### 2.2 Data collection

The semi-structured interviews followed an interview guide (Supplementary File 1) that explored participants' insights across three broad areas: (1) understanding of AMR and AMS; and the (2) current and (3) future state of community pharmacy's AMS involvement. The interview guide was drafted by K.L. and refined in consultation with H.S. and A.B. Clinical relevance of the content was assessed by K.L based on their experience as a pharmacist. Piloting of the interview questions occurred with one participant. As no changes were made following the pilot test, the results were included in the findings. The interview questions were broadly informed by the Theoretical Domains Framework (TDF),^13^ an intervention science framework which focuses on barriers and facilitators to behaviour change at the individual, cultural and environmental levels. Using the TDF to support development of the interview guide was considered appropriate given the study's focus on exploring the behavioural determinants influencing community pharmacy's AMS involvement. The TDF has also been used in a similar manner in other AMS-focused studies.^14^, ^15^, ^16^

K.L. conducted all the interviews *via* telephone or Microsoft Teams due to the COVID-19 pandemic restrictions that were in place during the time of data collection, and to minimise timing limitations, particularly for participants engaged in clinical practice. Consent to participate was verbally obtained ahead of the interview's commencement. This allowed for a proactive approach to confirming if a participant had questions prior to the interview starting, rather than the onus being on the participant to contact the research team. All interviews were audio-recorded and transcribed. No incentives were provided to participants.

### 2.3 Data analysis

The qualitative analysis approach used a combined method of inductive and deductive thematic analysis.^17^ Deductive analysis was guided by a coding framework that reflected the 14 TDF domains.^18^ Data were coded against the TDF domains where applicable, and inductive codes were assigned to data that were considered as not being categorised to the TDF domains. K.L. applied the coding framework across the transcripts, including identification of inductive codes, and multiple codes were applied, where appropriate. Results of the analysis were discussed with H.S. and A.O. in an iterative manner to support refinement of the coding framework, organizing into key themes and to reach consensus. The transcripts were analyzed on two levels. First, they were read for concepts that addressed the broader issue of attitudes and perceptions towards AMS in community pharmacy, and then secondly, more specifically at the level of implementation of AMS practices in community pharmacy. Analysis was facilitated using NVivo 12 Pro (QSR International Pty Ltd., 2018).

### 2.4 Ethics

Ethics approval for the study was obtained from the UNSW Human Research Ethics Advisory Panel G: Health, Medical, Community and Social (HC190119).

## 3 Results

### 3.1 Participant characteristics

Fifteen interviews were conducted with key informants. This encompassed personnel with experience in government roles relating to promoting AMS, non-government advocacy roles, clinical experience in the community or hospital setting, and pharmacy related research and academia. Many participants described previous or current professional experience across more than one of these settings; for example, having a non-government advocacy position while continuing to practice in community pharmacy.

### 3.2 Key themes

Thematic analysis identified four key themes which described key informants' insights on the perceived influences on community pharmacists' current and future involvement in AMS. (see Table 1).

**Table 1:** Themes, sub-themes and relevant theoretical domains.

Themes	Sub-themes	TDF
1. AMS perceived as a contextual rather than integrated concept		• Knowledge
2. Environmental factors influencing the relative ease and extent of AMS engagement		• Environmental context and resources
3. Professional relationships, timing and policing	GP-pharmacist interactions Timing of patient interactions	• Social role/identity
4. Perceived status of an antimicrobial prescription in validating and valuing a clinical interaction	Expected consultation outcome Commercial considerations	• Intentions • Beliefs about consequences

### 3.3 Theme 1: AMS perceived as a contextual rather than integrated concept

When asked to describe the meaning of the term ‘AMS’, most participants outlined AMS as the collection of activities that seek to reduce inappropriate use of antimicrobials.

#### 3.3.1 Understanding stewardship

However, some participants expressed that the term ‘stewardship’ was not commonly used in primary care and perceived that it was more closely associated with the hospital setting.

*I don*'*t think we would ever use the term ‘stewardship’ in dealing with patients or consumers. I think it*'*s an incredibly foreign concept. We even find that most people in primary care don*'*t understand it. [Interview #4]*

Reflecting on their clinical experience, one participant described needing to reframe the concept of stewardship, because of the perceived negative connotations of the term being associated with regulation, control, or cost-containment.

*So, the initial connotations when you talked about antimicrobial stewardship…, this is about not letting doctors prescribe what they want to prescribe and it*'*s about cutting cost. And so, we tried to put that positive spin on it in terms of no, it*'*s not necessarily about those things, those things might be an outcome of antimicrobial stewardship activities, but the primary driver is to promote quality use or optimal use of antibiotics in recognising that they are a precious resource… [Interview #5]*.

### 3.4 Theme 2: Environmental factors influencing the relative ease and extent of AMS engagement

Descriptions of the perceived influence of the environmental context on the relative ease and extent for AMS implementation, both at a health system level and within a community pharmacy environment was commonly raised.

Most participants, particularly those with clinical experience in a hospital environment, described assumptions on the relative ease to implement AMS within a hospital setting compared to primary care. Some participants described the presence of AMS accreditation standards, and the normalisation of multidisciplinary team care approach as being enabling features of the hospital environment which support a structured and consistent approach to AMS.

*Looking at, the challenges in primary care versus what happens in hospitals, hospitals have command and control, they have four walls, they have governance processes which it isn*'*t easy to replicate in primary care and so we*'*ve been dealing with the challenges of that. [Interview #4]*.

The perceived absence or difficulty in replicating these structural enablers within a primary care setting were raised as potential barriers to community pharmacists' AMS involvement.

*Now in hospital … they have antibiotic stewardship, and there*'*s a pharmacist there who looks at it, and they go on the wards and monitor and try to reduce antibiotic use… but it*'*s a controlled environment where everyone*'*s in the same room, you know, all talking to each other. I think in a hospital setting, a GP setting and an aged care setting the pharmacist can have the conversation with the prescriber and they*'*re part of that team. But to have you know, the pharmacist … ring[ing] up the local GP and questioning whether they*'*ve prescribed the right thing or not, the whole thing is not going to work. [Interview #7]*.

### 3.5 Theme 3: Professional relationships, timing and policing

Professional relationships and timing of interactions between pharmacists, GPs and patients were described.

#### 3.5.1 GP – pharmacist interactions

The nature of the professional relationship between a community pharmacist and the GP was often raised as both an enabler and barrier. Participants often reflected on personal clinical practice experiences where positive relationships and strong rapport with a local GP was conducive to supportive conversations about optimising antimicrobial use. This was often connected to views on a pharmacist's level of confidence in their clinical skills and judgement as the ‘medicines expert’ to raise concerns with a GP, and whether this respect for a pharmacists' medicines expertise was recognised. There was also a sense that the nature of a pharmacists' ability to communicate effectively was a critical dimension, with terms such as “diplomatic” and “questioning” used to describe an optimal approach to engaging with prescribers. Challenging professional relationships – described by one participant as ‘prickly’ [Interview #8] – were connected to descriptions of a diminished interaction between a pharmacist and a GP on AMS. For example, this included limiting interactions to raise safety concerns such as inappropriate dose or duration, rather than discussions on optimal prescribing.

*“Like with everything else, it's a relationship…. it*'*s having that collaborative relationship with the doctor to go hey is there a better option? I have concerns about this, as opposed to you know, you being the police. The pharmacist as being the police going - you've done it wrong again. Why have you done this? And then it just gets people offside.” [Interview #14]*.

### 3.6 Patient interactions

Participants' descriptions of community pharmacists' ability to influence AMS within their scope of practice appeared to be related to the timing of their interaction with a patient pre-or post-consultation with a GP.

The pre-consultation scenario was often described as community pharmacies performing a “triage” function, providing symptomatic treatment such as “cold and flu tablets” and subsequent referral, if required based on their professional judgement. This triage role was explored further when questioning participants on their views of Urinary Tract Infection Pharmacy Pilot – Queensland^19^ that was occurring during the time of this study. This pilot allowed trained community pharmacists to provide appropriate treatment which may have included antimicrobials, for uncomplicated urinary tract infections. Many participants expressed support for the trial, referencing that the presence of clinical guidelines and protocols to support community pharmacists in their decision making, including referral where required, was evidence of the profession respecting their scope of practice in contributing to patient care.

However, on balance, most participants described community pharmacists' AMS role as addressing the downstream effects of prescribing, in the post-consultation phase. This included references to ensuring the correct dose and duration of an antimicrobial had been prescribed and educating and reinforcing information provided by a prescriber on appropriate antimicrobial use. Some participants also described the lack of access to full clinical information as limiting their ability to provide an informed suggestion to a prescriber on an alternative antimicrobial, if warranted.

Striking a balance on how information is phrased when supporting a patient to make an informed decision about an antimicrobial prescription was also described. Tensions between suggesting that an antimicrobial may not be required to a patient within the scope of their professional judgement, without compromising the patient's trust in the information provided from their GP – which in turn, could affect the nature of future professional relationships were expressed. However, descriptions of a lack of clarity on whether this type of clinical intervention – either suggesting an alternative antimicrobial or suggesting a delay in dispensing a prescription as part of a ‘wait and see’ approach – would have professional indemnity implications was heard.

*“… you*'*re more likely to get in trouble for not doing something or withholding something than you are for doing something that is overly conservative in terms of overtreatment.” [Interview #5]*.

To a lesser extent, some participants connected AMS activities such as counselling and education provision, to community pharmacists' broad remit to promote the quality use of medicines. In this vein, AMS was perceived as part of ‘business as usual’ rather than a separate or specialised function. However, this was often qualified regarding the nature of AMS activities. For example, providing patient information and engaging in continuing professional development was provided as an example of part of usual community pharmacists' functions, where engaging in workshops or outreach activities were considered additional.

*“One could argue that quality use of medicines, and any antimicrobial stewardship activity in the community pharmacy is the natural course of the dispensing process.”[Interview #12]*.

### 3.7 Theme 4: Perceived status of an antimicrobial prescription in validating and valuing a clinical interaction

Many participants perceived that patients saw the issuing of an antimicrobial prescription as evidence which validated a clinical condition and connected this to the value of the professional interaction.

*“Once a doctor actually prescribes an antibiotic, the right to prescription, it the patient's mind that's validated that yeah, antibiotics are necessary.”[Interview #11].*

### 3.8 Expected consultation outcome

Related to this, participants expressed perceived challenges in influencing the dispensing of an antimicrobial prescription with a patient because of this validation notion and connected this to a need to engage GPs to promote AMS at the ‘source’. However, some participants reflected that symptomatic treatment, such as cold and flu tablets, could be written on a prescription – with this offered as a potential solution to supporting optimal use of antimicrobials, but continuing to meet patient's expectations on receiving a prescription as an expected outcome of a consultation.

*“…in some ways the clinicians involved in that scenario I think, might think, this [AMS] will take me more time, it*'*s too complex a concept to unpack with this patient, that antibiotics are not warranted here, so I just really need them to move on and get out, so again, often reaching for the prescription pad in this general practice consult, is the signal that the consult is reaching a conclusion and the patient can get up and leave now.”[Interview #4]*.

Some participants expressed views that some patients actively seek antimicrobial prescriptions as a consultation outcome. One participant suggested that some patients were aware of optimal scenarios in which an antimicrobial prescription could be obtained – “…they actually know how to game the system so you know, again they might know to go to the big corporate practice or because they'll get what they want, or they know to go on in towards the end of the day, when the doctor's resilience is less” [Interview #4]. To a lesser extent, participants currently practising in community pharmacy described a perceived influence of cultural factors as impacting consumers' understanding of AMS, and therefore expectation of an antimicrobial prescription, when reflecting on the patient demographics in their local communities. There was a sense that the seriousness of AMS could be diminished, with patients asking for antimicrobials because of cultural influences such as the availability of antimicrobials over the counter in some countries and viewing antimicrobials as a standard solution for “when they're feeling a bit off” [Interview #9].

### 3.9 Commercial considerations

At the community pharmacy level, there were perceptions that commercial considerations both in the immediate and future term, influenced the dispensing of an antimicrobial prescription. In the immediate term, some participants, particularly those in government or non-government sectors, expressed that the nature of community pharmacy as a business resulted in antimicrobial prescriptions representing a ‘sale’. Therefore, financial implications on dispensing may take precedence. Connected to this, some participants described that should a patient perceive the interaction to be negative because of a denied or delayed antimicrobial prescription, this could negatively impact a patient's ongoing and future custom of their community pharmacy.

*“…why would I bother making that effort if the prescribers aren*'*t on top of it yet… like they should be the ones to, to clean up their game first before I take on the risk of you know, having difficult conversations with patients who will just walk out of my shop into another pharmacy and that has financial implications…where a patient is less like that with their prescriber.” [Interview #2]*.

## 4 Discussion

The study's findings suggest that key stakeholders from Australia's community pharmacy sector perceive that promotion of community pharmacists' AMS involvement may better resonate when framed within their existing role in promoting the quality use of medicines. Further, the ability to adopt an antimicrobial guardian or gatekeeper role is influenced by the timing of their interaction with a patient either prior to, or post-consultation with a GP. However, the results also suggest that community pharmacists' potential and actual role AMS could be challenged or even restricted due to lack of access to completeness of clinical information, and perceived consequences from a clinical and professional engagement perspective.

Familiarity with the idea of AMS was evident among the participants, however the reference to ‘stewardship’ appeared to be perceived as a contextual rather than integrated concept. The prominence and requirement of stewardship within Australian hospitals may be a reason for why participants connected ‘stewardship’ as being restricted to this setting. Renaming AMR and its related terminology such as AMS in line with health communication principles, was suggested by Krockow (2020)^20^ to seek to promote awareness and better articulate the meaning of these terms to the public. This concept could be extended to community pharmacists, by reframing stewardship within a more familiar quality use of medicines narrative as suggested by some participants.

Participants made few references to needing to increase community pharmacists' clinical knowledge of AMR and AMS. Rather, participants focused on interpersonal and communication skills as key enablers to establishing a collaborative relationship with prescribers to enable discussions which support optimal antimicrobial use. These views reflect other findings that indicate effective interpersonal and communication skills, presence of mutual trust and appreciate, and recognition of the competence of the other health professional as supportive elements for collaboration between general practitioners and pharmacists.^21^^,^^22^ Broom and colleagues (2015) qualitative study of Australian pharmacists' accounts of antibiotic decisions in hospitals characterised ‘antibiotics-as negotiation’^23^ - highlighting the importance of pharmacists' negotiation and bargaining skills, particularly in seeking to shift prescribers' perceptions of pharmacists as playing a policing rather than supporting role within the context of AMS. Establishing conductive factors for GP and community pharmacist collaboration may be influenced by the extent to which traditional dynamics and relationships between the professions exist,^24^ with this challenge not merely limited to AMS. Addressing these dynamics at a provider level, rather than a profession wide level as suggested by Dobson and colleagues (2009) may be an appropriate way to build an interprofessional approach to practice^25^ in support of community pharmacists' AMS involvement.

Descriptions of the perceived influence of community pharmacy in AMS – as guardian and gatekeeper – appeared to encompass more activities with a patient pre-consultation with a GP compared with post. Participants' descriptions of the “triage” function of community pharmacists may be reflective of the profession's increasing role in managing minor ailments, such as common

colds^26^^,^^27^ which are commonly encountered by community pharmacists in developing^28^ and developed countries.^29^ Connecting community pharmacists' management of coughs and colds as minor ailments within the context of AMS has been highlighted by some of the sector's peak bodies, such as the UK's 10.13039/501100003924Royal Pharmaceutical Society.^26^ This AMS contribution may be further supported by specific protocols for community pharmacy management of coughs and colds to support consistency in service delivery and to achieve optimal health outcomes, particularly in circumstances where community pharmacists' may be able to provide over the counter antimicrobials for treatment of conditions such as influenza^30^ or uncomplicated urinary tract infections.^31^^,^^32^

Participants' reflections on patient demand as a perceived driver for antimicrobial prescribing concurs with previous study findings that have investigated this from a general practitioner perspective.^33^, ^34^, ^35^, ^36^ These findings indicate the presence of knowledge-practice dissonance, where general practitioners, in response to perceived patient demand, prescribe antimicrobials even when not medically indicated.^34^^,^^37^ Managing expectations in relation to antimicrobial prescribing has long been a focus of various AMS campaigns, where the onus is often on the consumers to be educated as to when an antimicrobial may be required.^38^

However, our findings suggest from a community pharmacy perspective, that it is the mere issuing of a prescription that may be valued by patients, as supporting evidence of a diagnosed condition. The use of prescription pads to recommend non-antibiotic solutions focused on symptomatic relief as an AMS strategy has been promoted by the US Centers for Disease Control and Prevention,^39^ the UK's National Health Service (NHS)^40^ and explored in research studies.^36^^,^^41^ The findings from Lee and colleagues' (2020) mixed methods study exploring the use of a ‘viral prescription pad’ as an educational tool for AMS in primary health care in Canada suggested that this resource was useful to guide discussions with patients focusing on symptomatic relief, rather than antibiotic use, for viral conditions.^41^ There is an opportunity to further investigate the use of this ‘non-prescription’ type resource in the Australian context, as our study's findings suggest that this type of resource may be effective in supporting community pharmacists, in addition to general practitioners, in their interactions with consumers about AMS.

While these findings have provided insights from key stakeholders in the Australian community sector on community pharmacy's AMS involvement, there are some limitations. The small sample size means that these findings may be limited in their generalisability. However, participants were drawn from across the sector in line with the study's objectives, and saturation of themes was achieved.

Using the TDF in developing the interview guide and analysis provided a structured approach to exploring the constructs relating to behaviour in line with the study's objective. However, data collection and analysis were not limited in its focus on the TDF domains. Through applying inductive and deductive analysis with the TDF as a basis, we consider that this has allowed broad exploration of participants' perspectives.^42^

Participants also described patient perceptions on community pharmacists' AMS involvement, which were not explored with a patient population in this study as this was outside the study's scope. Exploring the concordance between the sector's perception and patient's expectation of community pharmacists' AMS involvement is an opportunity for future research. Additionally, while this study was conducted during the COVID-19 pandemic which may have influenced participants' responses, discerning this was out of scope, and may be an area for future investigation.

## 5 Conclusion

Enabling community pharmacists to be antimicrobial gatekeepers and guardians requires the establishment and maintenance of collaborative partnerships between general practitioners and community pharmacists in the primary care setting as a key foundational element. While awareness of AMR and AMS exists in the Australian community pharmacy sector, there is an opportunity to frame this involvement within the broader quality use of medicines agenda to promote awareness and drive involvement. Further, the community pharmacy sector's movement towards professional service delivery, particularly with minor ailments, presents an opportunity to highlight the connections between these services and AMS.

## Declaration of Competing Interest

None.

## Appendix A Supplementary data

Interview guide Image 1
