# Supplementary material for: Community pharmacists as antimicrobial guardians and gatekeepers – A qualitative study of the perspectives of pharmacy sector stakeholders
Source: Explor Res Clin Soc Pharm. 2022 Dec 13;9:100212. doi: 10.1016/j.rcsop.2022.100212 (PMC9793303; doi:10.1016/j.rcsop.2022.100212)
Supplement: Supplementary file 1 — Interview guide [file mmc1.docx]

# Interview Guide – Stakeholders

| **Question** | | **Framework Alignment (TDF)** |
| --- | --- | --- |
| 1 | Could you tell me a little bit about your current role and how it interacts with the community pharmacy sector? |  |
| **Section 1:** **Antimicrobial resistance and antimicrobial stewardship** | | |
| 2 | Can you tell me a bit about what you know about antimicrobial resistance?   1. How do you think antimicrobial resistance comes about? 2. What do you think are the consequences of antimicrobial resistance? | Knowledge  Belief about Consequences |
| 3 | What does the term ‘antimicrobial stewardship’ mean to you?   1. How do you think stewardship is achieved? 2. Who do you think should be involved in antimicrobial stewardship? 3. Why is antimicrobial stewardship important? | Knowledge  Social/Professional Role and Identity  Belief about Consequences |
| 4 | Why do you think pharmacists are included in antimicrobial stewardship programs and initiatives?   1. What skills or knowledge helps pharmacists be involved in antimicrobial stewardship? | Social/Professional Role and Identity  Belief about Capabilities |
| **Section 2: Current state of community pharmacy in antimicrobial stewardship** | | |
| 5 | Antimicrobial stewardship has been described as a collective set of strategies to improve the appropriate use of antibiotics, as well as minimise the adverse effects of antibiotic use associated with resistance, toxicity and cost.  To what extent do you think community pharmacy is currently involved in antimicrobial stewardship?   1. How are community pharmacists involved?   *Probes*   - *Education and training* - *Influencing prescribing decisions* - *Promoting infection prevention and control initiatives (e.g. hand hygiene)*  1. Who else is involved in promoting antimicrobial stewardship? | Environment Context and Resources  Social Influences |
| 6 | To what extent do you think the general public currently seeks management of common infections in community pharmacy?   1. Why do you think this occurs?   *Probes*   - *How is self-care advice seen within scope of practice?* - *Cross-over with related topics, e.g. infection prevention and control* | Environment Context and Resources  Knowledge  Skills |
| 7 | What enables or supports community pharmacists being involved in promoting the effective antimicrobial stewardship practice?  *Probes*   - *Policy directives* - *Education and training* - *Role in primary health care team* | Belief about Capabilities  Environment Context and Resources |
| 8 | What barriers or challenges are there to community pharmacists being involved in promoting effective antimicrobial stewardship practice?  *Probes*   - *Policy directives* - *Education and training* - *Role in primary health care team* | Belief about Capabilities  Environment Context and Resources |
| 9 | Do you believe there are any factors impacting the safe and effective use of antibiotics from the perspective of:   1. General Practitioners 2. Patients | Environment Context and Resources  Skills  Knowledge |
| 10 | Do you think that promoting effective use of antibiotics is part of the role of a community pharmacist?   1. To what extent do you think this currently occurs? | Memory, Attention and Decision Processes  Belief about Capabilities  Skills  Social Influences |
| 11 | How effective do you think community pharmacists currently are in promoting the safe and effective use of antibiotics?   1. How do you know that community pharmacists are effective?   *Probes*   - *Peer feedback, clinical audits* - *Surveillance mechanisms* | Behavioural regulation  Intentions |
| **Section 3: Future state of community pharmacy involvement in antimicrobial stewardship** | | |
| 12 | In your opinion, how motivated are community pharmacists to contribute to antimicrobial stewardship?   1. Why or why aren’t they motivated? | Intentions  Optimism |
| 13 | What do you think would make community pharmacists more interested in being involved in antimicrobial stewardship?   1. How easy or difficult do you think it is to encourage this interest? | Reinforcement  Optimism |
| 14 | What in the health sector can stay the same to support community pharmacists being involved in antimicrobial stewardship?  *Probes*   - *Guidelines, frameworks* - *Funding mechanisms* - *Additional education and training* | Environment Context and Resources |
| 15 | What in the health sector needs to change to support community pharmacists in being involved in antimicrobial stewardship?  *Probes*   - *Guidelines, frameworks* - *Funding mechanisms* - *Additional education and training* | Environment Context and Resources |
| **End of Interview** | | |
